# Immunoreactivity of Plasminogen Activator Inhibitor 1 and Its Correlation with Dysmenorrhea and Lesional Fibrosis in Adenomyosis

**DOI:** 10.1007/s43032-021-00513-6

**Published:** 2021-03-08

**Authors:** Bingxin Yang, Nihao Gu, Shu Shi, Chen Zhang, Lan Chen, Jing Ouyang, Yu Lin, Feng Sun, Hong Xu

**Affiliations:** 1 International Peace Maternity and Child Health Hospital, School of Medicine, Shanghai Jiao Tong University, NO. 910, Heng-Shan Road, Xu-Hui Qu, Shanghai, 200030 China; 2 Shanghai Key Laboratory of Embryo Original Diseases, Shanghai, 200030 China

**Keywords:** Adenomyosis, PAI-1, Immunohistochemistry, Dysmenorrhea, Fibrosis

## Abstract

Adenomyosis is associated with dysmenorrhea, infertility, and lesional fibrosis. The pathogenesis of adenomyosis is still unclear. Plasminogen activator inhibitor 1 (PAI-1) plays important roles in pathological activities like tumor metastasis and endometriosis. Our objective was to investigate the expression and localization of PAI-1 in eutopic and ectopic endometrium with adenomyosis and in endometrium without adenomyosis. We also sought to determine the relationship between PAI-1 immunoreactivity and the severity of dysmenorrhea and the extent of lesional fibrosis in adenomyosis. PAI-1 expression was significantly higher in the ectopic endometrium of patients with adenomyosis than in both the eutopic endometrium of patients with adenomyosis and the endometrium of controls. Ectopic PAI-1 expression correlated positively with dysmenorrhea visual analog scale (VAS) scores and the extent of lesional fibrosis in adenomyosis. High PAI-1 expression increased the likelihood of moderate to severe dysmenorrhea in adenomyosis. These results suggest that PAI-1 is involved in the adenomyosis-associated dysmenorrhea and lesional fibrosis, which provide a potential target in treating symptomatic adenomyosis.

**Supplementary Information:**

The online version contains supplementary material available at 10.1007/s43032-021-00513-6.

## Introduction

Adenomyosis is a common chronic gynecological disorder accompanied by progressive dysmenorrhea and infertility [1, 2]. It is characterized by invasion of the glandular epithelium and stroma into the uterine myometrium, together with hyperplasia and hypertrophy of the myometrium [3]. At present, the pathogenesis of adenomyosis mainly includes the theory of invagination and metaplasia [4]. It has been reported that inflammation and innervation might be the key factors involved in the pathogenic mechanism of adenomyosis-related dysmenorrhea [5]. Adenomyosis impairs fertility mainly through reduced endometrial receptivity [6], which is influenced by uterine anatomical distortions due to adhesions and fibrosis [7, 8]. Therefore, the identification of elements involved in adenomyosis-associated dysmenorrhea and fibrosis is critical.

Plasminogen activator inhibitor 1 (PAI-1), encoded by the *SERPINE1* gene, belongs to the family of serine protease inhibitors and is essential for reacting rapidly with both urokinase-type and tissue-type plasminogen activators (uPA and tPA, respectively) [9]. PAI-1 participates in physiological or pathological activities such as cell adhesion, migration, and invasion and even in tumor formation and metastasis [10]. Studies have reported that PAI-1 might promote angiogenesis and stabilize neovascularization [11, 12], and elevated PAI-1 levels contribute to inflammation and excessive matrix deposition [13]. Researchers recently reported that PAI-1 is highly expressed in endometriosis [14]. Although endometriosis and adenomyosis are proximate in histomorphology and ectopic growth of the endometrium, their clinical characteristics and pathogenesis are not identical [15]. Thus, the expression and cellular localization of PAI-1 in adenomyosis and its associated uterine biology remain to be elucidated.

To investigate the expression and localization of PAI-1 in endometria with and without adenomyosis and to determine the relationship between PAI-1 immunoreactivity and the severity of dysmenorrhea and the extent of lesional fibrosis in adenomyosis, immunohistochemistry experiment, Masson’s trichrome staining, and correlation analysis between adenomyotic PAI-1 expression and the severity of dysmenorrhea and the extent of lesional fibrosis were performed. Furthermore, we evaluated the expression of PAI-1 with the inverse probability weighting (IPW) method to identify its potential role in the risk of the severity of dysmenorrhea in adenomyosis.

## Materials and Methods

### Sample Collection

All samples were obtained from our hospital between 2018 and 2019, fixed in 10% buffered formalin, and routinely processed for paraffin embedding. This study was approved by the Ethics Committee of the International Peace Maternal and Child Health Hospital (NO. GKLW 2017-71). Forty-five specimens of the ectopic endometrium and homologous eutopic endometrium of patients with pathologically confirmed adenomyosis were collected after hysterectomy or adenomyomectomy as the study group. For controls, endometrial samples were collected from patients who underwent laparoscopy with cervical carcinoma in situ or benign ovarian cysts. For patients with cervical carcinoma in situ, the endometria were collected through curettage after hysterectomy. For patients with benign ovarian cysts, we aspirated the endometrium with a Pipelle catheter from parous patients who did not need fertility and had fully informed consent [16]. Patients in the control group were without any clinical history, signs, gynecologic, and sonographic examinations of adenomyosis, endometriosis, and myoma in the preoperative state, laparoscopic examination, and histology after the surgery [17].

The adenomyosis and control groups were both required to fulfill the same criteria as follows: childbearing women who had regular menstruation (lengths varied from 21 to 35 days). The study flow chart is shown in Supplemental Figure 1. Written informed consent was obtained from all participants. The visual analog scale (VAS) was used to evaluate the dysmenorrhea of patients [18]. According to whether changing sanitary pads <3, between 3 and 6 or >6 times per day, the amount of menses during menstruation was grouped into 3 classes: light, moderate, and heavy [17].

### Immunohistochemistry

Formalin-fixed, paraffin-embedded tissue blocks were used for immunohistochemistry. Serial 4-μm sections were obtained from paraffin-embedded tissue blocks, with the first resultant slide stained for hematoxylin and eosin (H&E) to confirm pathologic diagnosis and the subsequent slides stained for PAI-1. Adenomyosis was defined by the ectopic endometrial glands and stroma at least one lower-power field of view (approximately 2–3 mm) away from the endometrial-myometrial junction [19]. Sections were rehydrated through graded alcohol and rinsed in distilled water. Antigen retrieval was conducted by microwave heating in citric saline for 15 min. Then, H_2_O_2_ and goat serum (10%) were used to deactivate endogenous peroxidase and block nonspecific binding, respectively. After that, samples were incubated with a PAI-1 rabbit polyclonal antibody (bs-6562R, Bioss, China, dilution 1:400) in a wet box at 4 °C overnight, followed by incubation with the secondary antibody. PBS was used as a negative control. DAB and hematoxylin were used to stain the sections. A series of 3 to 5 random views were captured by microscopy (Leica, DM2000). The number and intensity of positive cells were analyzed by Image-Pro Plus 6.0 (Media Cybernetics, Inc.) to evaluate the mean optical density (MOD), as reported previously [5, 20]. Briefly, the immunohistochemistry parameters measured in the view included the following: (a) integrated optical density (IOD); (b) total stained area; and (c) MOD, which was defined as MOD = IOD/S, equivalent to the staining levels in all positive cells. The immunoreactivity level of PAI-1 staining referred to the MOD values.

### Masson’s Trichrome Staining

Masson’s trichrome staining was used to detect collagen fibers in tissue samples. Tissue sections (4 μm, paraffin embedded) were deparaffinized in xylene, rehydrated in a graded alcohol series, and then soaked in Bouin’s solution at 37 °C for 2 h. Bouin’s solution was made with 75 mL of saturated picric acid, 25 mL of 10% formalin solution (v/v), and 5 mL of acetic acid. Tissue sections were stained using a Masson’s trichrome staining kit (RIBIOLOGY, Shanghai, China). The areas of the collagen fiber layer stained blue relative to the entire portion of the ectopic implants were calculated by Image-Pro Plus 6.0 and taken as the extent of lesional fibrosis as previously reported [21].

### Statistical Analysis

All values are presented as the mean (SD) or median (IQR) for continuous data according to whether the values were distributed normally assessed by the Shapiro-Wilk test. Pearson’s *χ*^2^ test or Fisher’s exact test was used to compare the proportions for categorical variables when appropriate. The comparison of distributions of continuous variables between two groups was made using Student’s *t* test or the Mann-Whitney *U* test, while distributions of continuous variables among three or more groups were analyzed using one-way *ANOVA* or the Kruskal-Wallis test. When both variables were continuous or at least one variable was ordinal, we used Pearson’s or Spearman’s rank correlation coefficient to evaluate correlations between them.

To assess the impact of the MOD value of PAI-1 on the severity of dysmenorrhea in adenomyosis, we calculated the odds ratio (OR, 95% CI) by binary logistic regression separately [22]. The interaction of the PAI-1 MOD value and extent of lesional fibrosis was evaluated. IPW, designed to minimize confounding in observational studies, is a statistical method widely applied in epidemiological studies [23] and has been used in IVF studies [24, 25]. In this study, we used the IPW method to better estimate the risk of moderate to severe dysmenorrhea among women with higher PAI-1 expression. All analyses were performed with R statistical software version 3.6.2 (packages MatchIt, reportReg, MASS). A *P* value less than 0.05 was considered statistically significant.

## Results

### Clinicopathologic Data

The characteristics of the adenomyosis and control groups and the statistical significance of the difference in these characteristics between the two groups are listed in Table 1. Eighty-five women were included in this study: 45 with adenomyosis (adenomyosis group) and 40 without adenomyosis (control group). The adenomyosis and control groups were comparable in age and menstrual phase, yet women in the adenomyosis group tended to have more pregnancies (*P*=0.043). It seems that the severity of dysmenorrhea in the adenomyosis group was more severe than that in the control group. Among the 45 patients with adenomyosis, 7 (15.6%), 13 (28.9%), 10 (22.2%), and 15 (33.3%) complained of having none, mild, moderate, and severe dysmenorrhea, respectively, and patients in the adenomyosis group had a higher median VAS score than patients in the control group (*P*<0.001).

**Table 1:** Characteristics of women in the adenomyosis and control groups and significant differences between the two groups

Characteristic	Control group (*n* = 40)	Adenomyosis group (*n* = 45)	*P* value
Age (years; median [IQR])	45 (7)	45 (6)	0.301
Menstrual phase			
Proliferative	24 (60.0%)	23 (51.1%)	0.411
Secretory	16 (40.0%)	22 (48.9%)	
Gravidity			
0	5 (12.5%)	2 (4.4%)	0.043
1	10 (25.0%)	3 (6.7%)	
2	12 (30.0%)	18 (40.0%)	
≥3	13 (32.5%)	22 (48.9%)	
Parity			
0	5 (12.5%)	5 (11.1%)	0.346
1	32 (80.0%)	36 (80.0%)	
2	1 (2.5%)	4 (8.9%)	
3	2 (5.0%)	0 (0.0%)	
Severity of dysmenorrhea			
None	29 (72.5%)	7 (15.6%)	<0.001
Mild	11 (27.5%)	13 (28.9%)	
Moderate	0 (0.0%)	10 (22.2%)	
Severe	0 (0.0%)	15 (33.3%)	
VAS score for dysmenorrhea (median [IQR])	0 (1)	5 (7)	<0.001
Amount of menses			
Light	18 (45.0%)	20 (44.4%)	0.788
Moderate	11 (27.5%)	10 (22.2%)	
Heavy	11 (27.5%)	15 (33.3%)	

Data are presented as the median (IQR) or number (percentage)

*VAS* means visual analog scale; *IQR* means interquartile range

### PAI-1 Expression Levels in the Control Endometrium, Eutopic Endometrium, and Ectopic Lesions

**Fig. 1:**
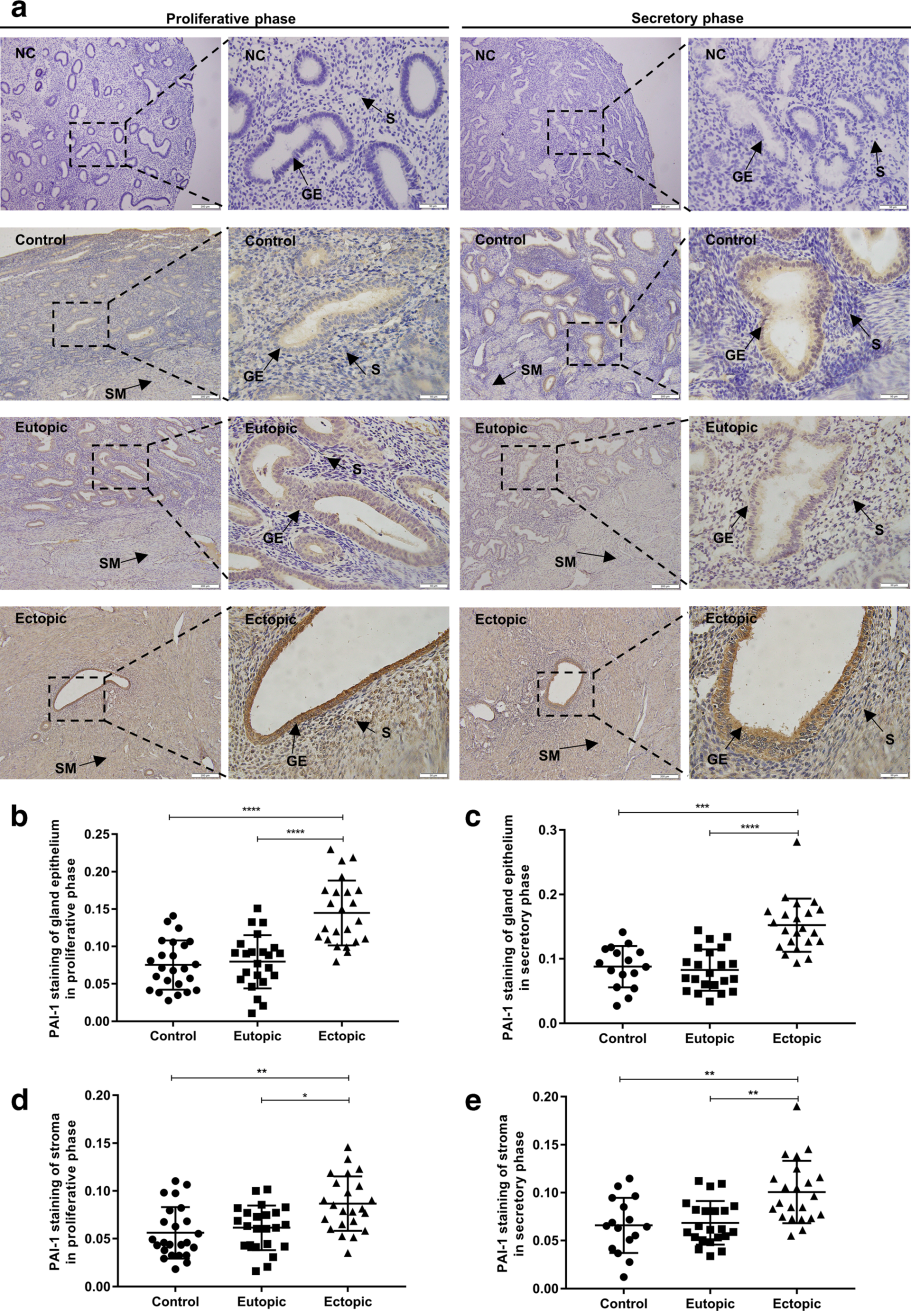
Immunohistochemical staining for PAI-1. **a** Representative photomicrographs of the immunohistochemical analysis of PAI-1 in ectopic and paired eutopic endometria with adenomyosis and control endometrium without adenomyosis. Black arrows indicate the localization of the glandular epithelium (GE), stroma (S), and smooth muscle (SM). **b** and **c** Quantitative analysis of the MOD values of PAI-1 immunoreactivity in the glandular epithelium among control, eutopic, and ectopic endometria in the proliferative phase and secretory phase, respectively. **d** and **e** Quantitative analysis of the MOD values of PAI-1 immunoreactivity in the stroma among control, eutopic, and ectopic endometria in the proliferative phase and secretory phase, respectively. *, *P*<0.05; **, *P*<0.01; ***, *P*<0.001; ****, *P*<0.0001. NC, negative control; GE, glandular epithelium; S, stroma; SM, smooth muscle

PAI-1 was stained throughout the uterine tissue, not only in the endometrium but also in the myometrium and vessels. PAI-1 immunoreactivity was more intense in the glandular epithelium than in the stroma, and intense PAI-1 staining was found mainly in the membranes and cytoplasms of glandular epithelial cells in the ectopic endometrium (Fig. 1a.

The MOD value of PAI-1 staining of the glandular epithelium in the ectopic endometrium with adenomyosis was significantly stronger than that in the eutopic endometrium with adenomyosis and the normal endometrium without adenomyosis (for the proliferative phase, both 푃 < 0.0001; for the secretory phase, 푃<0.0001 and 푃=0.0001, respectively) (Fig. 1b and c). Regarding the endometrial stroma, we observed a similar pattern: PAI-1 expression in the ectopic stroma was also higher than that in the eutopic and control endometria (for the proliferative phase, 푃=0.0235 and 푃=0.0014, respectively; for the secretory phase, 푃=0.0011 and 푃=0.0012, respectively), but such an increase in the stroma was not as obvious as in the glands (Fig. 1d and e). Variation between the proliferative phase and secretory phase of PAI-1 immunoreactivity was not observed in the control, eutopic, or ectopic endometrium or in the glandular epithelium or stroma (for the control endometrium, *P*=0.2320 and *P*=0.2354, respectively; for the eutopic endometrium, *P*=0.7241 and *P*=0.3138, respectively; for the ectopic endometrium, *P*=0.5360 and *P*=0.1342, respectively) (Supplemental Figure. 2).

### Associations Between PAI-1 Immunohistochemistry and the Extent of Lesional Fibrosis or the Severity of Dysmenorrhea in the Adenomyosis Group

**Fig. 2:**
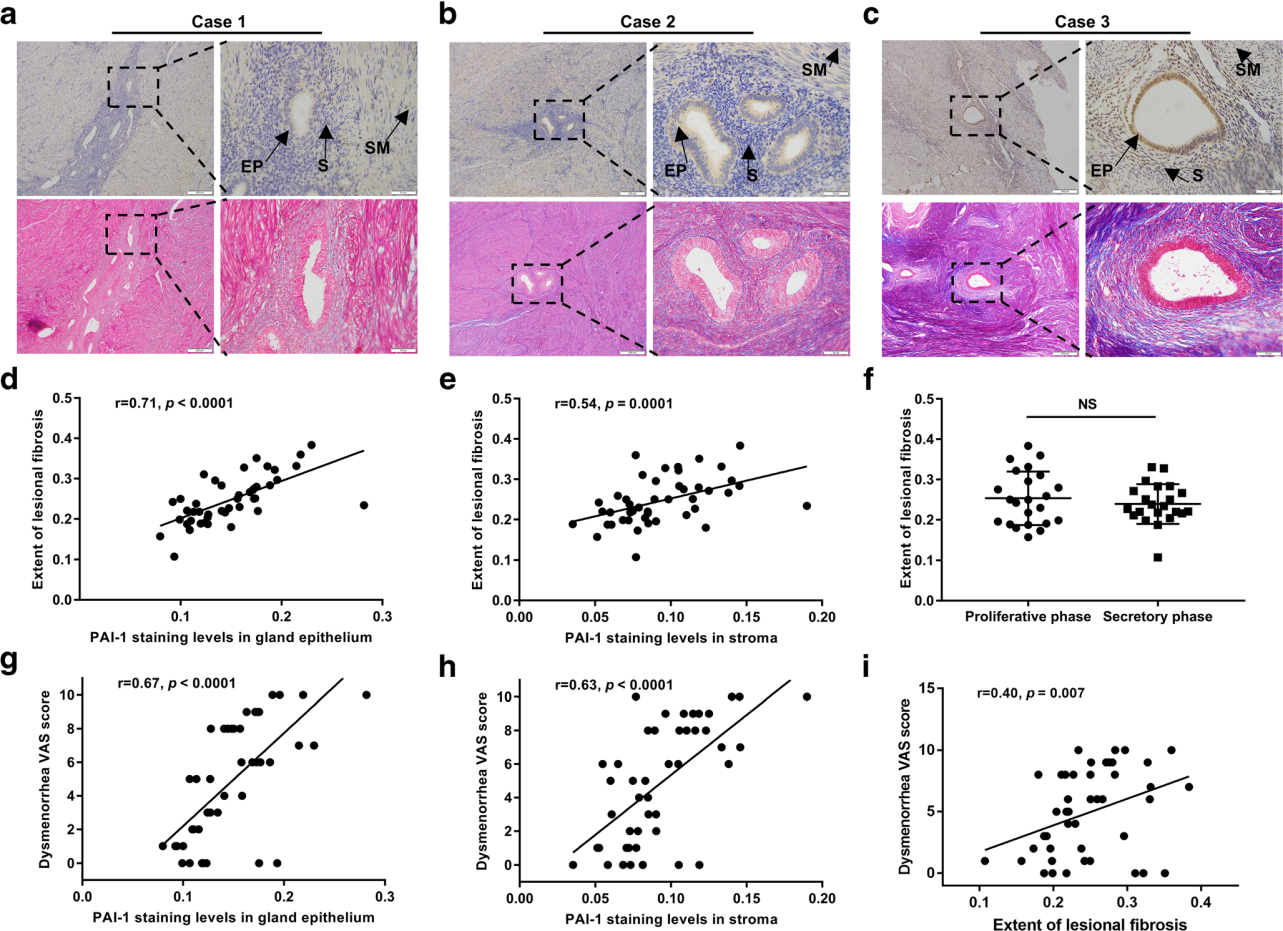
Associations among lesional PAI-1 immunoreactivity, the severity of dysmenorrhea, and the extent of fibrosis. **a**–**c** Masson’s trichrome-stained fibrosis and corresponding PAI-1 immunoreactivity in the biopsy samples derived from three representative cases. Black arrows indicate the localization of the glandular epithelium (GE), stroma (S), and smooth muscle (SM). Correlation of PAI-1 expression levels with the extent of lesional fibrosis in the glandular epithelium (**d**) and stroma (**e**); extent of lesional fibrosis between the proliferative phase and secretory phase (**f**); correlation between PAI-1 expression levels and the severity of dysmenorrhea, as measured by the VAS scores, in the glandular epithelium (**g**) and stroma (**h**); correlation of the extent of lesional fibrosis with dysmenorrhea VAS scores (**i**); the dashed line indicates a linear regression fit of the data. NS, *P*>0.05. *M*OD, mean optical density; GE, glandular epithelium; S, stroma; SM, smooth muscle

Representative images of Masson’s staining corresponding to specimens with different intensities of PAI-1 staining are presented (Fig. 2a–c). Correlation analysis showed that the extent of lesional fibrosis positively correlated with the ectopic staining levels of PAI-1 (*r*=0.71, *P*<0.0001; *r*=0.54, *P*=0.0001, respectively, in the glandular endometrium and stroma) (Fig. 2d and e). A difference in the extent of fibrosis between the proliferative phase and secretory phase was not observed in the ectopic endometrium (*P*=0.4199) (Fig. 2f). In addition, increased ectopic PAI-1 staining levels in the glandular epithelium and stroma were significantly correlated with increased dysmenorrhea (*r*=0.67, *P*<0.0001; *r*=0.63, *P*<0.0001, respectively) (Fig. 2g and h). Nevertheless, an association between eutopic PAI-1 expression and dysmenorrhea levels had no statistical correlation (*r*=−0.04, *P*=0.7778; *r*=0.22, *P*=0.1488, respectively). Interestingly, the severity of dysmenorrhea was also slightly correlated with the extent of fibrosis in adenomyotic lesions (*r*=0.40, *P*=0.007) (Fig. 2i). In contrast to dysmenorrhea and fibrosis, there was no statistical correlation between ectopic PAI-1 expression and the volume of menstrual flow in the glandular epithelium and stroma (*r*=0.12, *P*=0.4389; *r*=−0.02, *P*=0.8898, respectively).

### Effects of Adenomyotic PAI-1 Status and the Severity of Dysmenorrhea in Adenomyosis

To further analyze the associations between PAI-1 MOD values and the severity of dysmenorrhea, patients with adenomyosis were categorized into two groups by the VAS score: absent to mild dysmenorrhea (VAS=0–3 cm) or moderate to severe dysmenorrhea (or VAS=4–10 cm) [22]. Binary logistic regression analysis, which included age, menstrual phase, gravidity, and parity, confirmed that the PAI-1 MOD value in either anatomical compartment was an independent predictor of moderate to severe dysmenorrhea (*P*=0.002, aOR=1.07, 95% CI=1.22–2.39; *P*=0.005, aOR=1.64, 95% CI=1.16–2.33, respectively) (Table 2). Furthermore, we divided the PAI-1 MOD values into two groups according to the median MOD value among ectopic endometria with adenomyosis. Binary logistic regression indicated that the group with a higher MOD value had a greater risk of moderate to severe dysmenorrhea in the glandular epithelium or stroma, and the risk after stratification was also higher (*P*=0.002, aOR=48.17, 95% CI=4.17–557.01; *P*=0.003, aOR=13.64, 95% CI=2.36–78.78). The results were similar, while systematic differences were addressed with the use of the IPW method (Table 2). Moreover, the interaction of the extent of lesional fibrosis and the PAI-1 MOD value in the epithelium or stroma was not significant (Table 2), indicating that the association of the PAI-1 MOD value with moderate-to-severe dysmenorrhea in adenomyosis is similar in all cases of lesional fibrosis.

**Table 2:** Risk of moderate to severe dysmenorrhea in adenomyosis by binary logistic regression models according to the PAI-1 MOD value of the ectopic endometrium in patients with adenomyosis (*n*=45)

	Absent to mild dysmenorrhea vs. moderate to severe dysmenorrhea			
	Unadjusted OR (95% CI)	*P* value	Adjusted^a^ OR (95% CI)	*P* value
PAI-1 expression in the epithelium^c^	1.62 (1.23–2.15)	0.001	1.071 (1.22–2.39)	0.002
PAI-1 MOD value < 0.145	Ref.		Ref.	
PAI-1 MOD value ≥ 0.145	17.94 (3.88–83.09)	<0.001	48.17 (4.17–557.01)	0.002
IPW^b^, PAI-1 expression in the epithelium				
PAI-1 MOD value < 0.145	Ref.		Ref.	
PAI-1 MOD value ≥ 0.145	14.48 (5.06–41.46)	<0.001	52.09 (8.51–318.47)	<0.001
PAI-1 expression in the stroma^c^	1.72 (1.22–2.42)	0.002	1.64 (1.16–2.33)	0.005
PAI-1 MOD value < 0.086	Ref.		Ref.	
PAI-1 MOD value ≥ 0.086	10.29 (2.53–41.75)	0.001	13.64 (2.36–78.78)	0.003
IPW^b^, PAI-1 expression in the stroma				
PAI-1 MOD value < 0.086	Ref.		Ref.	
PAI-1 MOD value ≥ 0.086	8.33 (3.16–21.99)	<0.001	14.23 (4.05–49.97)	<0.001
Fibrosis				
Fibrosis by PAI-1 in the EP* interaction*	0.97 (0.93–1.01)	0.970	0.99 (0.94–1.03)	0.507
Fibrosis by PAI-1 in the ST* interaction*	0.96 (0.91–1.02)	0.150	0.97 (0.92–1.02)	0.234

*OR* means odds ratio; *PAI-1* means plasminogen activator inhibitor 1; *EP* means epithelium; *ST* means stroma

^a^OR adjusted for age, menstrual phase, gravidity, and parity

^b^Data weighted by the inverse of the probability of age, menstrual phase, gravidity, and parity

^c^Data are shown as the risk of moderate to severe dysmenorrhea in adenomyosis for each hundred numerical increases in the PAI-1 MOD value

## Discussion

Several studies have revealed that PAI-1 is dysregulated in numerous pathological states, such as metabolic syndrome, vascular diseases, fibrosis, and different kinds of cancer [26]. In a recent study, endometriotic PAI-1 expression in deep infiltrating endometriosis (DIE) was significantly higher than that in the eutopic endometrium [14]. Our study illustrated high PAI-1 expression in the ectopic endometrium with adenomyosis for the first time, and its expression is not regulated by the menstrual cycle. The increase in PAI-1 expression in the ectopic endometrium might contribute to limiting the invasive potential and proteolytic activity of the ectopic endometrium in adenomyosis, contributing to the deposition of fibrin and extracellular matrix components and finally forming regional lesions in the myometrium, which was similarly reported in advanced stages of endometriosis [27, 28]. Combined with our previous results of the higher *SERPINE1* mRNA level in adenomyosis [29], the increasing levels of both mRNA and protein of PAI-1 in the ectopic endometrium suggested the vital role of PAI-1 in the pathogenesis of adenomyosis.

Determining what causes dysmenorrhea in adenomyosis may help identify a therapeutic target for relieving this debilitating symptom. It was noted that elevated tissue factor (TF) immunoreactivity was related to the increased severity of dysmenorrhea in adenomyosis [30], and TF in nonhematopoietic cells induced adipocyte PAI-1 expression, indicating the regulation of PAI-1 by TF signaling [31]. In addition, Alotaibi et al. illustrated that PAI-1 expression in endometriosis was associated with dysmenorrhea by promoting inflammation [32]. Our results showed that increased PAI-1 expression in the ectopic endometrium was significantly associated with more severe dysmenorrhea in adenomyosis, which may indicate that PAI-1 could be a vital therapeutic target for adenomyosis-associated dysmenorrhea.

The profibrotic nature of lesions was investigated in recent studies of endometriosis [33]. Several factors may contribute to fibrosis in endometriosis: high expression levels of α-SMA and ADRB2 positively correlate with more extensive fibrosis, while E-cadherin expression levels negatively correlate with the fibrotic content [5]. Nonetheless, few molecules have been examined to explore their correlation with the extent of lesional fibrosis in adenomyotic lesions. To the best of our knowledge, PAI-1 regulates fibrinolytic activity, wound healing, and matrix remodeling at physiological levels, but in diseases, PAI-1 could be caused by some cytokines and thus result in tissue fibrosis [13]. Multiple reports have reflected the profibrotic role of PAI-1 in the lung, liver, and kidney [34]. Likewise, our finding that PAI-1 expression in the ectopic endometrium was positively correlated with the extent of lesional fibrosis first revealed a molecule that may be associated with fibrosis of the adenomyotic lesion in adenomyosis.

Although the research has reported that endometrial PAI-1 is significantly high in patients with essential menorrhagia in the menstrual phase [35], we found no statistical correlation between ectopic PAI-1 expression and the amount of menses in patients with adenomyosis, regardless of in proliferative or secretory phases.

We further revealed the expression of PAI-1 in different cell types, including glandular epithelial and stromal cells of the endometrium. In addition, we treated PAI-1 expression as an independent variable for moderate to severe dysmenorrhea in adenomyosis by binary logistic regression. One limitation is that the study was cross-sectional, and the influence of other variables on the results could not be excluded; therefore, we used the IPW statistical method to adjust for confounding factors.

In summary, this study initially revealed significantly high PAI-1 expression in the ectopic endometrium and its positive correlation with the severity of dysmenorrhea and the extent of lesional fibrosis in adenomyosis. The MOD value of adenomyotic PAI-1 expression was used to evaluate moderate to severe dysmenorrhea in adenomyosis. These findings suggest that PAI-1 could be mediated in the pathogenesis of adenomyosis and its associated dysmenorrhea and lesional fibrosis; thus, it might be a potential target in treating symptomatic adenomyosis.

## Supplementary Information

ESM 1 (PDF 171 kb)

## Data Availability

The data sets used and/or analyzed during the current study are available from the corresponding author on reasonable request.
