# Supplementary material for: Immunoreactivity of Plasminogen Activator Inhibitor 1 and Its Correlation with Dysmenorrhea and Lesional Fibrosis in Adenomyosis
Source: Reprod Sci. 2021 Mar 8;28(8):2378–86. doi: 10.1007/s43032-021-00513-6 (PMC8289782; doi:10.1007/s43032-021-00513-6)

Supplemental Figure 1. Study flow chart

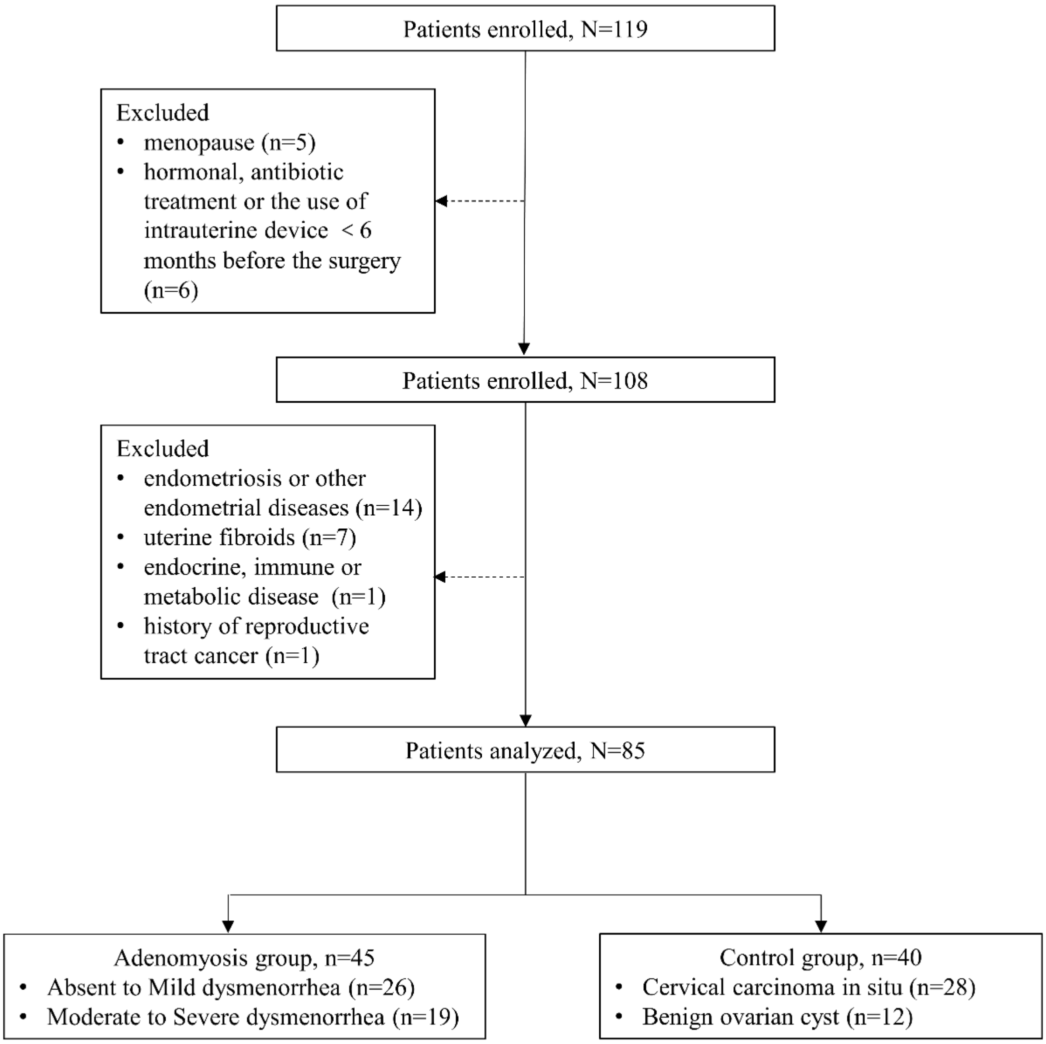

Supplemental Figure 2. Quantitative analysis of the mean optical density (MOD) of PAI-1 between the proliferative phase and secretory phase

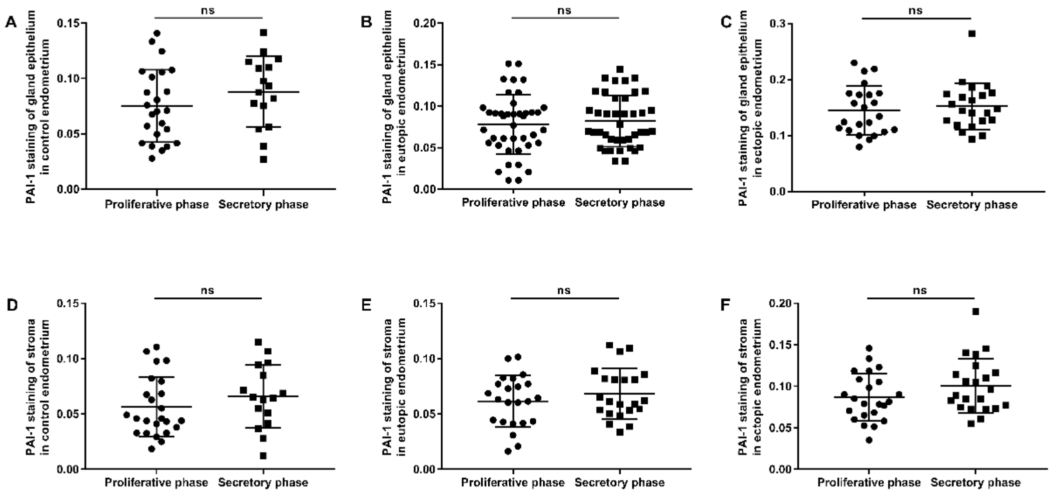

Supplement: Supplementary file 1 — (PDF 171 kb) [file 43032_2021_513_MOESM1_ESM.pdf]
